# A commensal symbiotic interrelationship for the growth of *Symbiobacterium toebii *with its partner bacterium, *Geobacillus toebii*

**DOI:** 10.1186/1756-0500-4-437

**Published:** 2011-10-24

**Authors:** Kwang Kim, Joong-Jae Kim, Ryoji Masui, Seiki Kuramitsu, Moon-Hee Sung

**Affiliations:** 1 Department of Biological Sciences, Graduate School of Science, Osaka University, Osaka 560-0043, Japan; 2 BioLeaders Corporation, 559 Yongsan-dong, Yuseong-gu, Daejeon 350-500, Korea; 3 Department of Advanced Fermentation Fusion Science and Technology, Kookmin University, Seoul 136-702, Korea; 4 RIKEN SPring-8 Center, 1-1-1 Kouto, Sayo-cho, Sayo-gun, Hyogo 679-5148, Japan; 5 Department of Biological Sciences, University of Calgary, Calgary, Alberta, T2N 1N4, Canada

**Keywords:** *Symbiobacterium toebii*, *Geobacillus toebii*, Bacterial symbiosis, Growth-supporting factor, Commensalism

## Abstract

**Background:**

*Symbiobacterium toebii *is a commensal symbiotic thermophile that absolutely requires its partner bacterium *Geobacillus toebii *for growth. Despite development of an independent cultivation method using cell-free extracts, the growth of *Symbiobacterium *remains unknown due to our poor understanding of the symbiotic relationship with its partner bacterium. Here, we investigated the interrelationship between these two bacteria for growth of *S. toebii *using different cell-free extracts of *G. toebii*.

**Results:**

*Symbiobacterium toebii *growth-supporting factors were constitutively produced through almost all growth phases and under different oxygen tensions in *G. toebii*, indicating that the factor may be essential components for growth of *G. toebii *as well as *S. toebii*. The growing conditions of *G. toebii *under different oxygen tension dramatically affected to the initial growth of *S. toebii *and the retarded lag phase was completely shortened by reducing agent, L-cysteine indicating an evidence of commensal interaction of microaerobic and anaerobic bacterium *S. toebii *with a facultative aerobic bacterium *G. toebii*. In addition, the growth curve of *S. toebii *showed a dependency on the protein concentration of cell-free extracts of *G. toebii*, demonstrating that the *G. toebii*-derived factors have nutrient-like characters but not quorum-sensing characters.

**Conclusions:**

Not only the consistent existence of the factor in *G. toebii *during all growth stages and under different oxygen tensions but also the concentration dependency of the factor for proliferation and optimal growth of *S. toebii*, suggests that an important biosynthetic machinery lacks in *S. toebii *during evolution. The commensal symbiotic bacterium, *S. toebii *uptakes certain ubiquitous and essential compound for its growth from environment or neighboring bacteria that shares the equivalent compounds. Moreover, *G. toebii *grown under aerobic condition shortened the lag phase of *S. toebii *under anaerobic and microaerobic conditions, suggests a possible commensal interaction that *G. toebii *scavengers ROS/RNS species and helps the initial growth of *S. toebii*.

## Background

Approximately 99% of microorganisms in natural environments have been estimated as being uncultivable using current methods. Technical difficulties arise because the constitution of microbial communities remains largely unknown [1-3], and thus adequate understanding of their diversity remains elusive. Until now, no detailed research had examined interspecies communication amongst uncultivable microorganisms even though progressive analyzing methods such as fluorescence *in situ *hybridization - microautoradiography [4,5], stable-isotope probing [6,7], and isotope array [8] had been reported to monitor a community of countless microorganism group in special environmental samples. Information was limited to how growth of uncultivable cells is affected by that of other microorganisms, how these cells progress to death or a dormant state and how growth is induced. At the same time, however, important findings concerning the commensal symbiotic and parasitic relationships between bacteria and eukaryotes, as well as cell-to-cell communication within a bacterial species, were being made [9-12].

Recent research on uncultivable microorganisms has led to a new cultivation method to isolate such microorganisms from nature. As a representative report, Kaeberlein and colleagues reported an *in situ *cultivation method to construct a simulated natural environment using a diffusion chamber that can provide certain growth-associated factors originating from adjacent microorganisms [13]. As similar approaches for the study of uncultivable microorganisms, we had developed a simple cultivation method by using either cell-free extract (CFE) or culture supernatant of its cognate bacteria to supply certain growth-supporting factor (GSF) for a commensal uncultivable bacterium [14]. Using the direct cultivating method with CFE of partner bacterium, we successfully isolated an intriguing bacterium from compost in the natural environment. The taxonomic analysis using 16S rDNA sequence revealed that it belonged to *Symbiobacterium*, in which growth absolutely depends on co-existence of the helper bacterium, *Geobacillus *species [14,15]. Studies of the distribution and diversity of *Symbiobacterium *species using competitive quantitative PCR, terminal restriction fragment length polymorphism and denaturing gradient gel electrophoresis revealed that these bacteria are widespread in natural environments, including compost, animal faeces, feeds and soils [16-19].

In our previous reports, we showed that *Symbiobacterium *species absolutely require an unidentified *Symbiobacterium toebii *growth-supporting factor (*syto*GSF) from partner bacteria for their growth and showed no growth under artificial culture conditions without *G. toebii *[14,15,20]. Despite the development of pure cultivation methods for *Symbiobacterium*, the isolation and cultivation of this genus remain difficult due to the lack of information on rigorous culture conditions, its growth mechanisms and symbiotic relationships with partner bacteria. In addition, the fundamental question as to what the *syto*GSFs are also remains debatable. Despite extensive studies on independent culture systems, the low reproducibility of axenic cultivation of *S. toebii *demonstrates that it has a complicated relationship with the partner bacterium, *G. toebii*, and also demands strict culture conditions for stable proliferation.

Moreover, no study has been reported to the relationship between two commensal bacteria for the initial growth of *S. toebii *and to the conditions for production of *syto*GSFs from *G. toebii*, meanwhile the study about the characters of *syto*GSFs produced by partner bacteria were relatively focused on the commensal symbiosis between *S. toebii *and *G. toebii *[20]. Understanding this representative relationship for commensal symbiosis between two different genera will give insights into basic information to elucidate the growth mechanism of other uncultivable bacteria which absolutely need GSF for its growth. We hope that this report could contribute to the cultivation of novel bacteria contained in 99% of the uncultivated bacteria of nature.

## Results

### *syto*GSFs are produced in almost all growth stages of *G. toebii*

In mixed cultivation of *S. toebii *with *G. toebii*, we previously reported that growth of *S. toebii *initiated and promoted after co-culture with *G. toebii *reached a stationary phase [14]. However, when *syto*GSFs are produced from partner bacteria is remaining controversial. To clarify this fundamental question, we proposed two hypotheses: (i) *G. toebii syto*GSFs are produced and secreted into the culture medium during the stationary phase similar to secondary metabolites, or (ii) *syto*GSFs are constitutively produced at every growth stage and are accumulated and released into the culture medium at stationary phase by cell lysis.

**Figure 1:**
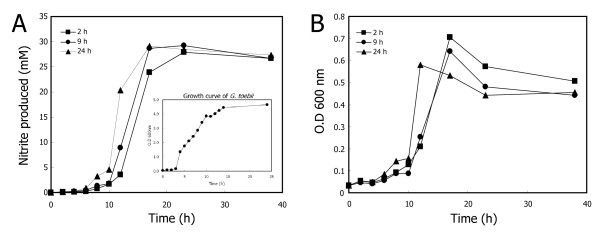
**Comparison of *S. toebii *growth curves and dependence on cell-free extracts from various *G. toebii *growth stages**. *S. toebii *was inoculated and cultured at 60°C in 100 ml PEPN broth under anaerobic conditions with 200 μg ml^-1 ^GtoCFE prepared from various *G. toebii *growth stages. *G. toebii *was cultured for 2 h (lag phase, squares), 9 h (exponential phase, circles) and 24 h (stationary phase, triangles), and then CFEs were prepared. The *G. toebii *growth curve (as measured by optical density at 600 nm) is shown in the inset (panel A). *S. toebii *growth was monitored by nitrite production (A) and optical density at 600 nm (B).

To define the production phase of *syto*GSFs from *G. toebii*, we investigated growth-supporting activity using the same amount of cell-free extracts of *G. toebii *(GtoCFEs) extracted from each of three different growth phases of *G. toebii*, the lag phase (2 h cultivation), mid-exponential phase (9 h) and stationary phase (24 h). Nitrite production and optical density were used to assess the growth of *S. toebii *in response to each GtoCFE. Figure 1 shows that all three GtoCFEs had similar effects on the growth of *S. toebii*. The effects of GtoCFEs prepared from lag and exponential phase cells showed similar doubling times and optimal nitrite production to the stationary phase-extracted GtoCFEs, suggesting that *G. toebii *constantly produced *syto*GSFs throughout almost all growth stages.

### Growth of *S. toebii *depends on the concentration of GtoCFEs

In Figure 1, the result of growth-supporting activity against the growth phase-dependent GtoCFEs was consistent with the second hypothesis and suggested that a low cell mass of *G. toebii *at the lag phage produced a small amount of *syto*GSFs. We consider that the extended lag phase of *S. toebii *in mixed cultivation with *G. toebii *[14] caused by insufficient amounts of *syto*GSFs leads to exponential growth of *S. toebii *when *G. toebii *reached stationary phase and that this bacterium requires a certain concentration of *syto*GSFs for its stable proliferation.

**Figure 2:**
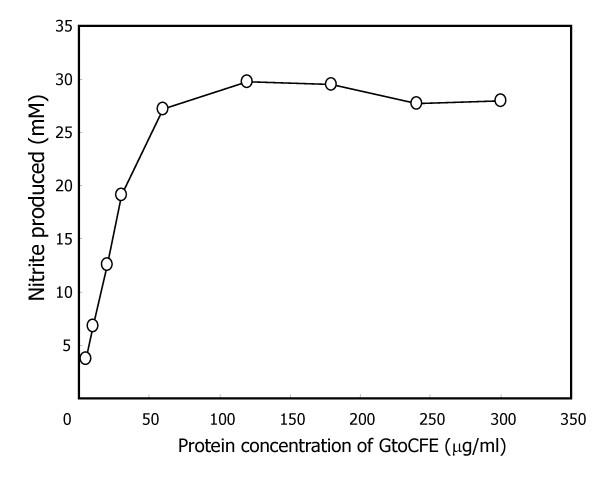
**Growth of *S. toebii *depends on the concentration of GtoCFE**. *S. toebii *was cultivated at various concentrations of GtoCFE in 10 ml PEPN broth at 60°C in an anaerobic jar with a GasPak™ Plus Anaerobic System Envelope for 18 h. The *x*-axis indicates the final protein concentration of the supplied GtoCFE in the culture broth. *S. toebii *growth was monitored by nitrite production.

**Figure 3:**
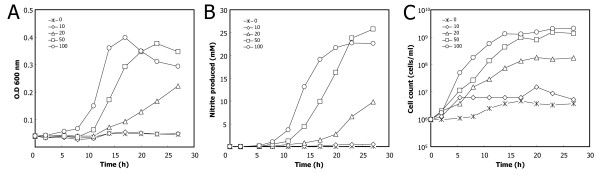
**Growth curve of *S. toebii *with respect to GtoCFE concentration**. One milliliter of anaerobically 12h-cultured *S. toebii *was inoculated and cultured at 60°C in 100 ml PEPN broth in a N_2 _gas-flushed anoxic cultivation system with 0, 10, 20, 50 or 100 μg ml^-1 ^GtoCFE. The growth of *S. toebii *was monitored by measuring the optical density at 600 nm (A), concentration of nitrite produced (B) and cell counts (C). Asterisks, without GtoCFE (negative control); circles, 10 μg ml^-1^GtoCFE; triangles, 20 μg/ml^-1^; diamonds, 50 μg ml^-1^; squares, 100 μg ml^-1^.

To investigate the effect of the concentration of *syto*GSFs required for stable growth, we examined the maximum growth rate of *S. toebii *in the presence of GtoCFE protein concentrations ranging from 5.0 μg ml^-1 ^to 300 μg ml^-1 ^(Figure 2). After 18 h of cultivation of *S. toebii *under anaerobic conditions, optimal growth was achieved with more than 60 μg ml^-1 ^protein of the GtoCFE. With 30 μg ml^-1 ^GtoCFE, only half-maximal growth was achieved, indicating that the growth rate of *S. toebii *depends on the concentration of factors in the GtoCFE. In addition, we investigated growth curves of *S. toebii *against various GtoCFE protein concentrations by measuring nitrite production, optical density (600 nm) and viable cell counts to verify the growth-supporting effect of GtoCFEs (Figure 3). A high concentration of GtoCFE (e.g., ≥ 50 μg ml^-1^) supported rapid and optimal growth of *S. toebii *within 17 h, but 10 μg ml^-1 ^GtoCFE did not substantially enhance the growth. Compared to 100 μg ml^-1 ^GtoCFE, the growth rate and doubling time were significantly retarded in the presence of 20 μg ml^-1 ^GtoCFE. These results strongly suggest that *syto*GSFs have a role as nutrient-like compounds and *S. toebii *requires *syto*GSFs throughout its all growth phases.

### The culture conditions of *G. toebii *under different oxygen tension affect the growth of *S. toebii*

Figure 1 showed that *G. toebii *constantly produced *syto*GSFs throughout almost all growth phases. However, *G. toebii *was cultivated under aerobic conditions for both of this experiment and previous reports, even though *S. toebii *cannot grow under aerobic conditions [21]. Since anaerobic or microaerobic conditions are essential for a commensal interaction between these two bacteria, we doubted that *G. toebii *could produce *syto*GSFs under microaerobic or anaerobic conditions. We reported that *G. toebii *can grow under aerobic conditions [22], but we recently found it can also grow under anaerobic and microaerobic conditions with nitrate as an electron acceptor. The optical density (600 nm) of culture broth of *G. toebii *at early stationary phase under the microaerobic and anaerobic conditions showed about 0.6 and the growth of *G. toebii *under both conditions was almost parallel (data not shown). Based on this result, we were able to prepare GtoCFEs from anaerobically and microaerobically cultivated *G. toebii *and determined the growth-supporting effect for *S. toebii*.

**Figure 4:**
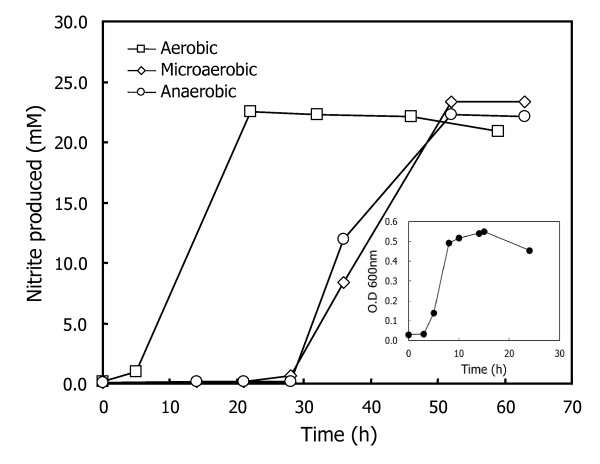
**Effects of GtoCFE prepared under various culture conditions on *S. toebii *growth curves**. *S. toebii *was cultured in a N_2 _gas-flushed anoxic cultivation system with 0.2 mg ml^-1 ^of various GtoCFEs (squares, prepared from aerobic culture; diamonds, prepared from microaerobic culture; circles, prepared from anaerobic culture). Each *G. toebii *was cultured for overnight under different culture conditions and the cell-free extract was prepared by following the methods section. Nitrite production was used as a measure of *S. toebii *growth. The *G. toebii *growth curve (as measured by optical density at 600 nm) under the anaerobic culture condition was shown in the inset.

Figure 4 shows that similar maximum growth can be achieved with all three supplements after 48 h cultivation. We noted that *S. toebii *cultured with GtoCFEs of aerobically cultured *G. toebii *had a 5-h lag time, whereas the lag times of *S. toebii *cultured with GtoCFEs prepared from microaerobically and anaerobically cultured cells were significantly extended to approximately 30 h. Moreover, all three GtoCFEs showed similar growth-supporting effects at the doubling time. This indicates that *G. toebii *can also produce *syto*GSFs under anaerobic and microaerobic culture conditions as well as under aerobic conditions. Furthermore, some factors contained in aerobically cultivated *G. toebii *dramatically support the initial growth of *S. toebii*.

### Reducing agent recovered the extended lag time of *S. toebii*

The results in Figure 4 suggest that GtoCFEs from microaerobically and anaerobically cultured *G. toebii *lack some factors necessary for adaptation and initial growth of *S. toebii *during the lag phase. Because *S. toebii *was quite sensitive to the oxygen tension, we further hypothesised that the CFE of aerobically cultured *G. toebii *contains plentiful machinery that eliminates toxicity against oxidative stresses by reactive oxygen and nitrogen species (ROS/RNS) in the culture medium. Furthermore, almost aerobic bacteria have enzymatic defense systems (e.g., superoxide dismutase, catalase and peroxidase) against oxidative stress [23,24].

**Figure 5:**
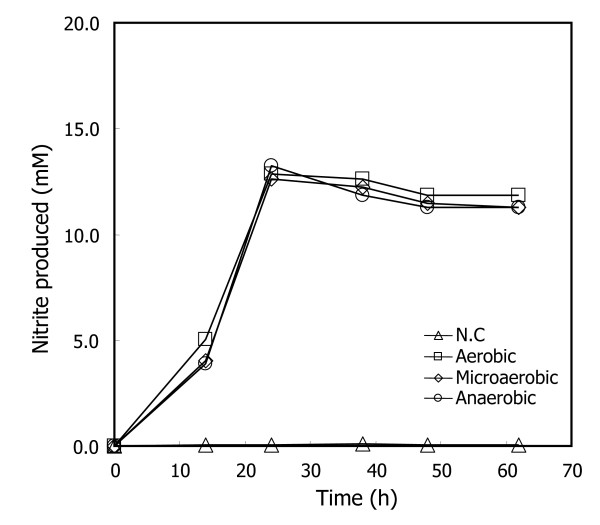
**The effect of L-cysteine as a reducing agent on *S. toebii *growth**. L-cysteine (0.025% final concentration) was added to 100 ml PEPN broth containing 0.2 mg ml^-1 ^of the same CFE prepared from microaerobically, anaerobically or aerobically cultured *G. toebii *with the experiment of figure 4. *S. toebii *was cultured in a N_2 _gas-flushed anoxic cultivation system at 60°C. Squares, with CFE prepared from aerobically cultured *G. toebii*; diamonds, with CFE prepared from microaerobically cultured *G. toebii*; circles, with CFE prepared from anaerobically cultured *G. toebii*; triangles, without CFE (as a negative control, N.C.). *S. toebii *growth was monitored by measuring accumulated nitrite in culture broth.

To investigate the effect of oxidative stress on lag time and examine whether an enzymatic defense system against ROS works in the GtoCFE supplied culture broth of *S. toebii*, we tested the effect of L-cysteine, a reducing agent known to alter the redox state of culture broth [25], in the culture broth that contained microaerobically or anaerobically prepared GtoCFEs for growth of *S. toebii*. The addition of 0.025% L-cysteine significantly shortened the lag time for GtoCFEs prepared with microaerobically or anaerobically cultured cells (Figure 5). Furthermore, the doubling times at the exponential growth stage were similar to those of aerobically prepared GtoCFEs. This result suggests that reduction of ROS/RNS by the enzymatic defense system in culture broth containing with aerobically cultivated GtoCFE could aid in constructing the initial conditions for growth of *S. toebii*.

## Discussion

The GtoCFE prepared from the lag and exponential growth phases supported similar growth of *S. toebii *to the GtoCFE prepared from the stationary growth phase (Figure 1). The doubling times were parallel at each exponential growth stage of *S. toebii*. Figure 4 also shows that almost all of the GtoCFEs prepared at various oxygen tensions caused similar optimum growth. Both results demonstrate that *syto*GSFs are constantly produced in *G. toebii*. This ubiquitous property of *syto*GSFs strongly suggests the possibility that *syto*GSFs may be essential material in *G. toebii *as well as in *S. toebii*. Since these two bacteria belong to different genera, we consider that *syto*GSFs may be vital and fundamental material in *Firmicutes *and *Proteobacteria*. Moreover, the CFE of *Escherichia coli*, *Bacillus subtilis *and *Geobacillus kaustophilus *also show similar growth-supporting effects to *S. toebii *[20]. This prevalent existence in many other bacteria and the putative vital property in both *G. toebii *and *S. toebii *strongly suggest that *S. toebii *may not produce certain essential compounds for growth and proliferation due to either the absence of genes encoding essential biosynthetic pathways or silence of expression of proteins to produce *syto*GSFs.

*S. toebii *cannot grow at atmospheric oxygen concentrations. In contrast, its partner bacterium *G. toebii *is a facultative aerobe that prefers aerobic over anaerobic growth conditions [22]. The discrepancy in oxygen tension for optimal growth of these two commensal bacteria raises interesting questions about how they interact directly and whether *G. toebii *also produces *syto*GSFs under microaerobic or anaerobic condition. The discrepancy between these bacteria also provides a clue to the extended lag times of *S. toebii *cultivated with the CFEs of anaerobically or microaerobically cultured *G. toebii*. Generally, the physiological response to oxidative stress of ROS has been well studied in bacteria, especially anaerobic bacteria. For example, *Clostridium perfringens *and *Desulfovibrio gigas *elicit such protective responses at early stages of growth [23,24]. Furthermore, L-cysteine is thought to be an effective reducing agent at less than 0.05% in a culture broth [25]. As predicted, the extended lag times of *S. toebii *cultivated with CFEs prepared from microaerobically and anaerobically cultured *G. toebii *were reduced by direct supplementation with 0.025% L-cysteine to the *S. toebii *culture broth. Because the doubling time of all three GtoCFEs during exponential growth phases did not change, we assume that L-cysteine reduced the existence of ROS/RNS in the culture medium at the initial growth stage for *S. toebii*. Therefore, we suppose that the ROS/RNS scavenger proteins in GtoCFEs of aerobically cultured *G. toebii *support growth of *S. toebii *during the lag phase. Genome sequence analysis of *G. kaustophilus*, which is closely related to *G. toebii*, revealed several genes encoding three superoxide dismutases (GK2288, GK2457 and GK2933), two catalases (GK1710 and GK3036), two peroxidases (GK1785 and GK2787) and an alkyl hydroperoxide reductase homologue (GK0477) [26].

**Figure 6:**
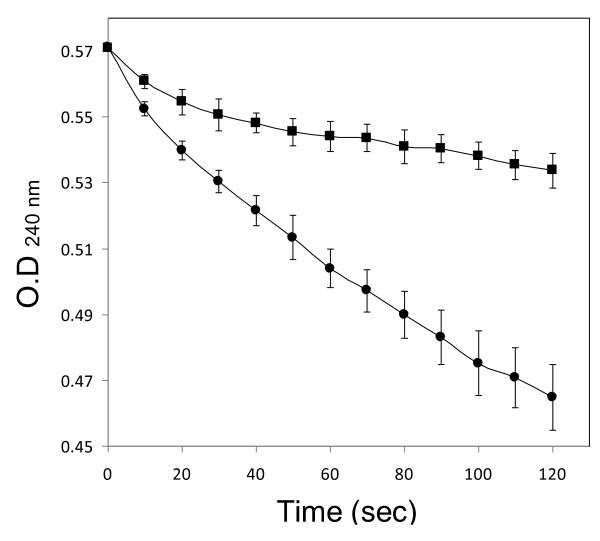
**The catalase and peroxidase activity in the GtoCFEs prepared from aerobically and anaerobically cultivated *G. toebii***. The breakdown H_2_O_2 _with aerobically prepared GtoCFE (closed circle) and anaerobically prepared GtoCFE (closed square) was directly monitored at absorbance 240 nm for 120 s under 25°C, respectively. The results represent the mean (± standard deviation) of five independent measurements.

To verify this hypothesis, we examined the activity of catalase and peroxidase in the cell-free extracts that prepared from aerobically and anaerobically cultivated *G. toebii*. In Figure 6, H_2_O_2 _in reaction mixture was rapidly diminished at the aerobically prepared GtoCFE, whereas the change of H_2_O_2 _concentration was gently decreased at the anaerobically prepared GtoCFE. This result supports that the elimination of ROS/RNS in the culture broth by those enzymes of helper bacteria promotes the growth of commensal symbiotic bacterium, *S. toebii *at its lag phase. These findings can also explain how *S. toebii *and *G. toebii *co-exist and how *G. toebii *supports the initial growth of *S. toebii *in environments such as compost and animal faeces. However, only construction of microaerobic and anaerobic conditions by *G. toebii *in the surrounding environment is not enough for the growth of *S. toebii *because this bacterium does not grow without special supplements such as GtoCFEs.

In the last decade, cell-signaling compounds were identified in *Euplotes raikovi *and *Micrococcus luteus*, which are pathogenic bacteria and the compounds are proposed to mediate communication within species to control cell population growth in response to various stimuli [27-29]. The growth of *S. toebii *appears to be stimulated and induced by *syto*GSFs from *G. toebii*. However, we propose that *syto*GSFs produced from *G. toebii *differ from the above quorum-sensing factors. *S. toebii *continuously required GtoCFE during all growth stages, and the growth rate was dependent on the concentration of *syto*GSFs in the GtoCFE (Figures 2, and 3). The observation that the doubling time was persistently extended with insufficient concentrations of *syto*GSF suggests a nutrient-like character for *syto*GSFs rather than a signaling character.

The most interesting issue about *S. toebii *study is what the *syto*GSF is. However, we couldn't characterize and purify the *syto*GSF in spite of extensive and exhaustive studies because of unstable cultivation results and low reproducibility of the growth of *S. toebii *in an artificial condition. Nevertheless, we suppose that properties of the growth-supporting factor for *S. toebii *and *S. thermophilum *are quite different because the growth-supporting factor for *S. thermophilum *(*syth*GSF) is existed in a culture supernatant whereas *syto*GSF is existed in a cell-free extract [20]. These two components also showed different biochemical properties such as molecular weight, heat stability and proteinase sensitivity [20]. Recently, Ueda *et al *suggested a possibility of syntrophic relationship between *S. thermophilum *and *Bacillus *strain S [30]. They focused carbonic anhydrase of the helper bacterium which reversibly converts CO_2 _to carbonate ion because no homolog of carbonic anhydrase was observed in genome sequence of *S. thermophilum*. They assumed that the carbonate ion and/or CO_2 _converted by carbonic anhydrase of the helper bacterium supports the growth of *S. thermophilum*. Additional supplement of high concentration of CO_2 _gas into the culture system promoted approximately 10% of the maximal growth of *S. thermophilum*. However, no significant growth of *S. toebii *was observed under the same CO_2 _concentration in the culture system without GtoCFE or culture supernatant of *G. toebii*. Taken together, we carefully concluded that the CO_2 _effect is not working for *S. toebii *and the growth-supporting factor for two *Symbiobacterium *is different although they belong to the same genus.

## Conclusion

The study of commensal interrelationships between two different bacterial genera based on the growth of *S. toebii *with various CFEs of *G. toebii *suggests that *syto*GSFs are ubiquitously expressed in *G. toebii *and may be essential compounds *Firmicutes *and *Proteobacteria*. Growth of *S. toebii *depended on the concentration of GtoCFE, indicating that *syto*GSF has nutrient-like characters rather than quorum-sensing character. Taken together, we suggest that *S. toebii *may not be able to produce *syto*GSFs due to silence or lack of certain biosynthesis machinery and uptakes the anonymous vital compound for growth from environment or partner bacteria which share the element. Additionally, the retarded lag phases of *S. toebii *by GtoCFEs of microaerobically or anaerobically cultivated *G. toebii *and the complementary effect of reducing agent, L-cysteine for the growth of *S. toebii *suggest a possible commensal interaction that ROS/RNS scavenger enzymes expressed in *G. toebii *under aerobic condition play an important role for the initial growth of *S. toebii*.

## Methods

### Bacterial strains, culture media and reagents

*S. toebii *SC-1 DSM 15906 (KCTC 0685BP) and *G. toebii *DSM 14590 were isolated from Korean hay compost (*toe-bi*). PEP broth was prepared by mixing 6 g KH_2_PO_4_, 2 g K_2_HPO_4_, 5 g polypeptone and 10 g yeast extract in 1.0 liter distilled water; the pH was adjusted to 7.0 with NaOH. NaNO_3 _was added to PEP broth at a final concentration of 30 mM to produce PEPN broth, and this was used to cultivate *S. toebii*. Agar plates were made by adding 20 g agar powder to 1.0 liter PEPN broth. Luria-Bertani (LB) broth for *G. toebii *was prepared by combining 10 g tryptone, 5 g NaCl and 5 g yeast extract in l.0 liter distilled water and adjusting the pH to 7.0. NaNO_3_, NaNO_2 _and kanamycin were purchase from Wako Pure Chemical Industries (Osaka, Japan). Sulphanilamide and *N*-(1-naphthyl)-ethylenediamine for nitrite quantification were purchased from Sigma-Aldrich (St. Louis, MO, USA). Yeast extract and Bacto™ Tryptone were purchased from Becton Dickinson (Sparks, MD, USA) and polypeptone and other chemicals for cultivation were obtained from Nacalai Tesque (Kyoto, Japan).

### Cultivation of *S. toebii *and *G. toebii*

An anaerobic culture system was constructed with a GasPak™ Plus Anaerobic System Envelope (BBL™ Cat. No. 271040; Becton Dickinson Microbiology Systems, Cockeysville, MD, USA), which kept the H_2_:CO_2 _gas ratio at 90:10, with < 0.2% O_2 _contained in an anaerobic jar (BBL™ GasPak 100™ Anaerobic System; Becton Dickinson Microbiology Systems). Anaerobic conditions were confirmed using Dry Anaerobic Indicator Strips (BBL™ Cat. No. 271051; Becton Dickinson Microbiology Systems). For other anoxic culture systems, 0.1- to 2.0-liter glass bottles were sealed with silicon packing and the air inside the bottles was purged with nitrogen gas for 1 min. Bottles were then charged with the same gas for an additional 1 min using a syringe and a filter (Millex^® ^GV 0.22 μm; Millipore, Cork, Ireland) at 0.2 Mpa. For microaerobic cultures, a CampyPak™ Plus Microaerophilic System Envelope (BBL™ Cat. No. 271045; Becton Dickinson Microbiology Systems) was used in place of the anaerobic system envelope in the same type of jar.

*S. toebii *was pre-cultured with 100 ml PEPN broth containing 100 μg ml^-1 ^GtoCFE prepared from aerobically cultivated *G. toebii *and 50 μg ml^-1 ^kanamycin in the anaerobic culture system at 60°C for 12 h. One percent of the culture broth was inoculated into the same culture medium which pre-incubated at 60°C over 1 h and cultured the broth anaerobically at the same temperature for a sufficient time depending on each experiment. The CFE of *G. toebii *was supplied into the culture broth according to the needs of each experiment. Aerobic cultivation of *G. toebii *was carried out with LB broth in an Erlenmeyer flask at 60°C overnight, or for the time required to prepare various GtoCFEs. *G. toebii *was cultivated microaerobically and anaerobically under the same conditions as *S. toebii*. The cultures were supplemented with 30 mM sodium nitrate as an electron acceptor for anaerobic respiration.

### Preparation of CFEs of *G. toebii*

To prepare culture condition-dependent GtoCFEs, *G. toebii *was cultivated at 60°C overnight in 1.0 liter of LB broth either with vigorous shaking for aeration or in a jar as described above without agitation for anaerobic and microaerobic cultivation. The cells were harvested at 7000 × *g *for 10 min at 4°C after 2 h, 9 h and 24 h to prepare GtoCFEs for lag, exponential and stationary growth stages, respectively. Pellets were washed twice with phosphate-buffered saline (PBS, pH 7.4) and resuspended in 20 ml PBS. The cells were disrupted ultrasonically (UD-210: output 8, duty 70; Tomy, Tokyo, Japan) three times for 10 min on ice with stirring. After centrifugation at 7000 × *g *for 20 min to remove cell debris, the crude extract was filter-sterilised with a 0.2-μm pore filter (Minisart-plus, 0.2 μm CA-membrane+GF-prefilter; Sartorius AG, Göttingen, Germany). This GtoCFE was used as a supplement for *S. toebii *cultures. The protein concentration of the GtoCFEs was estimated by the Bradford method using bovine serum albumin as a standard [31]. The concentration of GtoCFE ranged from 20 to 30 mg ml^-1^. To investigate the growth-supporting effect of *syto*GSFs, the *syto*GSFs contained in GtoCFEs were normalised to the protein concentration of the GtoCFEs.

### Measurement of bacterial growth

*S. toebii *growth was monitored by three independent methods: direct cell counting, optical density of the culture broth and accumulation of nitrite. Cells were counted in a 0.02-mm-deep bacteria counting chamber (Erma Co., Tokyo, Japan) under a phase-contrast microscope (BX40F; Olympus Optical Co. Ltd., Tokyo, Japan). Optical density (600 nm) was measured for each culture broth with a spectrophotometer (U-3000; Hitachi, Tokyo, Japan). The concentration of nitrite that accumulated in the medium after conversion from nitrate by *S. toebii *was measured using the modified colorimetric method described below. The growth of *G. toebii *was monitored by measuring the optical density at 600 nm.

### Quantification of nitrite

*S. toebii *converts nitrate to nitrite under nitrate-reducing conditions. Nitrite accumulates in the culture broth because it cannot be converted to N_2 _or NH_4_^+ ^[21]. Therefore, *S. toebii *growth can be monitored indirectly by measuring accumulated nitrite in the culture broth. A modified colorimetric method was used for this purpose: 20 μl of sulphanilamide solution (1% sulphanilamide in 0.6 N HCl) was added to 0.9 ml of serially diluted culture broth, and the mixtures were kept at room temperature for 4 min after vigorous agitation. Then, 20 μl of 0.1% *N*-(1-naphthyl)-ethylenediamine solution was added to each reaction, and the solutions were kept at room temperature in the dark for 30 min. Coupling of the nitrite-sulphanilamide complex to *N*-(1-naphthyl)-ethylenediamine caused the solution to turn pink. Absorbance at 543 nm was converted to nitrite concentration using a standard curve constructed by the known amounts of sodium nitrite.

### Catalase and peroxidase assay

*G. toebii *was cultivated by stationary phase at anaerobic system or aerobic system in LB broth with or without 30 mM sodium nitrate, respectively. Each protein concentration was normalised with PBS buffer as 20 mg/ml^-1 ^by Bradford method after cell lysis. The activity of catalase and peroxidase was measured by breakdown rate of H_2_O_2 _in a reaction mixture [32]. The cell-free extracts were further diluted to a hundredth with 50 mM potassium phosphate buffer (pH 7.0) and then 10 μl of the cell-free extract corresponding to 2.0 μg of total proteins was reacted with 10 mM H_2_O_2 _in 1.0 ml of 50 mM potassium phosphate buffer (pH 7.0) under 25°C. The change of H_2_O_2 _concentration directly monitored by UV spectrometer (U-3000; Hitachi, Tokyo, Japan) at absorbance 240 nm from 3 - 123 s after the onset of reaction.

## Abbreviations

CFE: cell-free extract; GtoCFE: cell-free extract of *G. toebii*; GSF: growth-supporting factor; *syto*GSF: growth-supporting factor for *Symbiobacterium toebii*; *syth*GSF: growth-supporting factor for *Symbiobacterium thermophilum*; ROS: reactive oxygen species; RNS: reactive nitrogen species.

## Competing interests

The authors declare that they have no competing interests.

## Authors' contributions

KK designed and interpreted data and wrote the manuscript. JJK assisted in the data acquisition. RM helped revising the manuscript. SK designed and supervised the analyses and corrected the manuscript. MHS designed project conception and helped to revise the manuscript. All authors read and approved the final manuscript.
